# The Circulating Fatty Acid Transporter Soluble CD36 Is Not Associated with Carotid Atherosclerosis in Subjects with Type 1 and Type 2 Diabetes Mellitus

**DOI:** 10.3390/jcm9061700

**Published:** 2020-06-02

**Authors:** Esmeralda Castelblanco, Lucía Sanjurjo, Maria Barranco-Altirriba, Mireia Falguera, Marta Hernández, Berta Soldevila, Maria-Rosa Sarrias, Josep Franch-Nadal, Juan Antonio Arroyo, José-Manuel Fernandez-Real, Nuria Alonso, Didac Mauricio

**Affiliations:** 1 Department of Endocrinology & Nutrition, Hospital de la Santa Creu i Sant Pau, IIB Sant Pau, 08041 Barcelona, Spain; esmeraldacas@gmail.com (E.C.); barmaria95@gmail.com (M.B.-A.); 2 Centre for Biomedical Research on Diabetes and Associated Metabolic Diseases (CIBERDEM), 08907 Barcelona, Spain; bsolde@hotmail.com (B.S.); josep.franch@gmail.com (J.F.-N.); 3 Innate Immunity Group, Health Sciences Research Institute Germans Trias i Pujol (IGTP), 08916 Badalona, Spain; lsanxurxo@gmail.com (L.S.); mrsarrias@igtp.cat (M.-R.S.); 4 Biomedical Research Institute of Lleida, University of Lleida, 25198 Lleida, Spain; mireiafalguera@hotmail.com (M.F.); martahernandezg@gmail.com (M.H.); 5 Primary Health Care Centre Cervera, Gerència d’Atenció Primaria, Institut Català de la Salut, 25200 Cervera, Spain; 6 Department of Endocrinology and Nutrition, University Hospital Arnau de Vilanova, IRBLleida, 25198 Lleida, Spain; 7 Department of Endocrinology & Nutrition, University Hospital Germans Trias i Pujol, IGTP, 08916 Badalona, Spain; 8 Centre for Biomedical Research on Liver and Digestive Diseases (CIBEREHD), 28029 Madrid, Spain; 9 DAP-Cat group, Unitat de Suport a la Recerca Barcelona, Fundació Institut Universitari per a la Recerca a l’Atenció Primària de Salut Jordi Gol i Gurina (IDIAPJGol), 08007 Barcelona, Spain; 10 Department of Internal Medicine, Hypertension and Vascular Risk Unit, Hospital de la Santa Creu i Sant Pau, 08041 Barcelona, Spain; jarroyo@santpau.cat; 11 Department of Medicine, Autonomous University of Barcelona, 08193 Bellaterra, Spain; 12 Department of Diabetes, Endocrinology & Nutrition, Hospital Dr Josep Trueta, IDIBGI, 17007 Girona, Spain; jmfreal@idibgi.org; 13 Centre for Biomedical Research on Physiopathology of Obesity and Nutrition (CIBEROBN), 17007 Girona, Spain

**Keywords:** atherosclerotic plaque, carotid atherosclerosis, sCD36, type 1 diabetes mellitus, type 2 diabetes mellitus

## Abstract

This study aimed to determine the association of fatty acid transporter plasma soluble cluster of differentiation 36 (sCD36) with subclinical carotid atherosclerosis (SCA). A cross-sectional study was conducted in 1023 subjects, 225 with type 1 diabetes (T1D), 276 with type 2 diabetes (T2D) and 522 who were nondiabetic. Carotid atherosclerotic plaque (CAP) presence was determined using B-mode carotid ultrasound imaging. sCD36 were analysed by ELISA, and CD36 surface receptor and mRNA expression were measured by flow cytometry and real-time PCR. Logistic regression models were used to evaluate sCD36 as a biomarker of SCA. Up to 376 (36.75%) participants had at least one CAP, 76 T1D, 164 T2D and 136 without diabetes, while the remaining 647 (63.25%) did not have any CAP. There were no differences in sCD36 between patients with and without CAP in T1D (*p* = 0.287) or T2D (*p* = 0.513). Although nondiabetic subjects with plaques had lower sCD36 levels than those without (*p* = 0.023), the multivariate models revealed no association of sCD36 with CAP in any of the three study groups. No differences were found in surface CD36 or CD36 mRNA expression between the patients with and without CAP. sCD36 is not associated with SCA in type 1 or type 2 diabetic or in nondiabetic subjects.

## 1. Introduction

Accelerated atherosclerosis is the primary cause of the increased cardiovascular morbidity and mortality associated with type 1 (T1D) and type 2 diabetes (T2D). Apart from other important cardiovascular risk factors, and although it has not been clearly established, there is some evidence that points to hyperglycemia as a contributing factor to the diabetes-associated macrovascular burden [1]. In addition, there is an urgent need to define new markers that contribute to the detection and prediction of atherosclerosis in patients with diabetes.

There are several factors that have been described to be involved in the pathogenesis of atherosclerosis in patients with diabetes. Among these, we find hyperglycemia, dyslipidemia and chronic inflammation [2]. In this sense, results obtained in studies performed in animal models of diabetes have suggested that atherosclerosis might be accelerated, in part, due to the synergistic effects of altered glucose and lipid metabolism [3]. On the other hand, there is solid evidence from experimental, clinical and epidemiological studies showing that hyperglycemia and its associated metabolic disturbances are linked to an increased inflammatory response that contributes through different pathways to the inflammatory response involved in the pathogenesis of the atherosclerotic process [2,3]. The cluster of differentiation 36 (CD36) is an 88-kDa transmembrane glycoprotein expressed in many cell types, and plays an essential role in lipid metabolism, angiogenesis and inflammation. This fatty acid transporter is related to several disease conditions such as cancer biology, insulin resistance, stroke, diabetes nephropathy and atherosclerosis [4,5]. Atherosclerosis develops as a result of lipid uptake by macrophages of the vascular wall, leading to the development of foam cells and smooth muscle cell proliferation [6]. In macrophages, the fatty acid transporter CD36 promotes atherosclerosis through the uptake of oxidized low-density lipoprotein (ox-LDL) and subsequent foam cell development [4], and the release of inflammatory mediators [5]. Previous studies demonstrated the involvement of CD36 in the progression of atherosclerosis, and also that deletion or blockage of the CD36-induced signalling pathway decreased the development of atherosclerotic lesions in mice [7,8].

For these reasons, CD36 has been proposed as a biomarker of future cardiovascular disease in diabetes with potential clinical applicability [9]. The circulating form of CD36 (sCD36) was reported to be related with metabolic syndrome for the first time in a small group of subjects: sCD36 was increased in T2D individuals compared with both lean (5-fold) and obese nondiabetic subjects (2- to 3-fold) [10]. These results were also reported by other studies from the one group [11,12,13]. However, other researchers have found an inverse relationship between sCD36 and several components of the metabolic syndrome [14,15]. In subjects with altered glucose tolerance, sCD36 was also reported to be related to markers of liver injury such as alanine aminotransferase (ALT) and aspartate transaminase (AST) [16]. Moreover, the fatty acid transporter sCD36 has been considered a potential biomarker of atherosclerosis given that, as mentioned before in animal models of atherosclerosis, absence of CD36 has been reported to result in a reduction of atherosclerosis [8]. Further, some authors have reported an association between higher sCD36 concentration and carotid intima-media thickness (cIMT), a surrogate marker of atherosclerosis [11,17]. Additionally, Handberg et al. reported that, in patients from the general population with internal carotid stenotic plaques, those who are symptomatic show higher sCD36 concentrations compared with those who are asymptomatic [18]. Finally, in another study in patients with stage 5 chronic kidney disease, higher sCD36 concentrations were associated with an increased risk of CV mortality [19].

In this study, we aimed to assess whether the circulating concentrations of sCD36 are associated with presence of carotid plaques in subjects with T1D, T2D, and without diabetes, all of them with normal kidney function and free of previous cardiovascular disease.

## 2. Experimental Section

### 2.1. Design and Study Population

This cross-sectional study included 1023 participants recruited from the University Hospitals Germans Trias i Pujol (Badalona, Spain) and Arnau de Vilanova (Lleida, Spain). Among them, 225 with T1D and 276 with T2D were selected from two previous studies [20,21]; 522 individuals were selected from a population-based study in our region (participants without diabetes on the basis of HbA_1c_ < 6.5% and glucose < 126 mg/dL) [22]. Further, 50 participants, 22 with and 28 without carotid atherosclerotic plaque, were enrolled at the University Hospital Germans Trias i Pujol to perform the sub-studies of flow-cytometric analysis and mRNA expression of CD36. Inclusion criteria for all the study participants included the following: age 20–85 years; free from established chronic kidney disease (glomerular filtration rate < 60 mL/min and/or urine albumin/creatinine ratio > 299 mg/g); absence of clinical cardiovascular disease or associated revascularisation procedures, such as cerebrovascular disease, coronary heart disease, or peripheral vascular disease (including the diagnosis of diabetic foot disease). A subject was classified as having hypertension or dyslipidaemia if he/she was taking medication for the given condition. Information about medication was obtained after a careful review of the clinical records. Anthropometric measures, including waist circumference, height, body weight, and sitting blood pressure, were obtained by standardized methods, and information about smoking habits and alcohol consumption was collected as previously described [20,21]. Blood samples were collected from subjects for all analyses in a fasting state. Standard laboratory methods were used to determine blood tests of glucose, HbA1c, creatinine, lipid profile, blood profile and ALT and urine tests (the latter only in diabetic subjects) [21]. Blood samples for sCD36 measurements were processed immediately after extraction and stored at −80 °C at institutional biobanks until analysed. Protocols for flow cytometry assays and real-time PCR started immediately after collection. Local ethics committees of University Hospitals Arnau de Vilanova (PI-13-095 and 12/2009), Germans Trias i Pujol (PI-15-147) and Primary Health Care University Research Institute (IDIAP) Jordi Gol (P12/043) approved the study, following the Declaration of Helsinki. Before inclusion, all participants provided written informed consent.

### 2.2. Carotid Ultrasound Imaging

Carotid B-mode ultrasound imaging was performed using a Sequoia 512 (Siemens, North Rhine, Westphalia, Germany) or LOGIQ E9 (General Electric, Wauwatosa, WI, USA) with 15-MHz linear-array probes. The presence of atherosclerotic plaques was identified using B-mode and colour Doppler examinations. The presence of atherosclerotic plaques at different carotid artery sites was defined as a cIMT ≥ 1.5 mm protruding into the lumen, according to the criterion given in the ASE Consensus Statement [23] and the Mannheim cIMT Consensus [24]. This criterion was used in two relevant studies showing the predictive value of atherosclerotic plaques for cardiovascular risk, the Atherosclerosis Risk in Communities study (ARIC) [25] and the Framingham Offspring Study [26]. Plaques were also classified as echolucent (lipid- and haemorrhage-rich plaques) or echogenic/mixed (fibrotic or fibro-fatty plaque and mixed when patients had both echolucent and echo-rich plaques), depending on plaque echogenicity on ultrasound examination, as previously described [27]. All ultrasound procedures were performed by experienced researchers following a standardized protocol.

### 2.3. Determination of sCD36 by ELISA

To assess the concentration of plasma sCD36, we used a commercial ELISA kit (Nordic BioSite, Täby, Sweden) following the instructions of the manufacturer. In brief, standards and samples, in appropriate dilutions, were incubated in duplicate for 2 h on the plate shaker at room temperature (RT). After washing four times, a detection antibody was added and incubated for 2 h on the plate shaker at RT. After four washes, a solution of streptavidin conjugated with horseradish peroxidase was incubated for 50 min again on the plate shaker protected from light at RT. After washing four times, a 50 μL substrate solution (TMB (tetramethylbenzidine)) was added, and this solution was incubated and protected from light for 20 min at RT. Then, 50 μL of stop solution was used to stop the colour development. Absorbance was read at 450 nm using SpectraMax 340PC384 (Molecular Devices, LLC, Sunnyvale, CA, USA). A log-log curve fit was used to analyse the results. The calibration was performed with recombinant human CD36 in a concentration range of 1.95–250 ng/mL. A value of 0.05 ng/mL was assigned to values of sCD36 lower than the detection limit (nondiabetic group, *n* = 64/522; T1D, *n* = 41/225 and T2D, *n* = 24/276). The intra-assay and inter-assay precision coefficients provided by the ELISA manufacturer were 4–6% and 8–12%, respectively.

### 2.4. Flow-Cytometric Analysis

The flow-cytometric analysis was designed to determine if there were differences in the expression of the surface receptor CD36 in circulating mononuclear cells between subjects with and without atherosclerotic plaques. This analysis included 50 subjects, 22 patients with and 28 without carotid atherosclerotic plaques. Blood was incubated with ammonium chloride (BD Pharm Lyse™, San Jose, CA, USA) for 10 min to lyse erythrocytes. The cells were washed with PBS and then incubated with monoclonal antibodies against CD36 (Miltenyi Biotec, Bergisch Gladbach, Germany), CD3 and CD14 (BD Biosciences, San Jose, CA, USA). Flow-cytometric analysis was performed on a Fortessa SORP flow cytometer (BD Biosciences, San Jose, CA, USA) using the sample acquisition and analysis software FACSDiva v6.2 (BD Biosciences, San Jose, CA, USA).

### 2.5. Real-Time PCR

In the 50 patients mentioned above, an analysis of CD36 mRNA expression was carried out to find differences between subjects with and without atherosclerotic plaque. After lysing, the erythrocytes with ammonium chloride (BD Pharm Lyse™, San Jose, CA, USA), the cells were washed with PBS and disrupted with QUIzol Lysis Reagent (Qiagen, Hilden, Germany). The mRNA Mini Kit (Qiagen, Hilden, Germany) was used to extract total mRNA. Total RNA (1 µg) was reverse-transcribed using the Transcriptor First Strand cDNA Synthesis Kit (Roche, Basel, Switzerland). Each reaction was then amplified in a LightCycler^®^ 480 PCR system using SYBR Green I Master (Roche, Basel, Switzerland). The CD36 primer pairs used in the reaction were forward primer 5′–›3′ (GAGAACTGTTATGGGGCTAT) and reverse primer 5′–›3′ (TTCAACTGGAGAGGCAAAGG). The expression level of glyceraldehyde 3-phosphate dehydrogenase (GAPDH) was used to normalise gene expression values before analysing the results.

### 2.6. Statistical Analyses

The R statistical software, version 3.3.1, and SPSS software (version 22, IBM, SPSS, Chicago, IL, USA) was used for all handling of data, statistical analysis and figure construction. The results of the quantitative measurements are expressed as mean (standard deviation) or median (interquartile range), while for qualitative variables, absolute and relative frequencies are used.

Analysis of variance (ANOVA), the Mann–Whitney test, or the Kruskal–Wallis test was used to determine the differences between patients with and without carotid atherosclerotic plaque in the T1D, T2D and control groups. The chi-squared test or Fisher’s exact test was used to evaluate the differences in qualitative variables. Tukey’s correction and Spearman’s rank correlation coefficient were used to account for multiple tests and correlations, respectively. A logistic regression model was used to determine the associations of variables with the presence of atherosclerotic plaque in every study group. In all these models, the variables of the bivariate analysis with a *p*-value < 0.1 and clinical relevance were used. The Hosmer–Lemeshow test evaluated in all models tested the goodness-of-fit assumption. Receiver operating characteristic (ROC) curves and DeLong’s test for correlated ROC curves were used to check the logistic regression models. Furthermore, multinomial logistic regression models were performed to determine the variables associated with the burden of carotid atherosclerotic plaque, one plaque or multiple plaques; patients without plaque were used as the reference. A *p* value < 0.05 was established as statistically significant.

## 3. Results

A total of 1023 individuals, 376 (36.75%) with and 647 (63.25%) without a carotid atherosclerotic plaque, were included in the study. In the T1D group (*n* = 225), 33.8% had atherosclerotic plaques; of 276 subjects with type 2 diabetes, 59.4% had plaques, and 26.1% of nondiabetic subjects (*n* = 522) had plaques. In the overall study population, the mean age was 51 ± 12.5 years, and up to 45.4% were men. In T1D, patients with at least one atherosclerotic plaque were older and had a higher proportion of tobacco exposure, hypertension, dyslipidaemia and antiplatelet treatment. Moreover, they had higher BMI (body mass index), SBP (systolic blood pressure) and ALT (alanine aminotransferase) than those without plaque. On the other hand, in the T2D group, patients with at least one atherosclerotic plaque were older and had a higher proportion of alcohol consumption, tobacco exposure and hypertension. Further, they had increased SBP and mean platelet volume (MPV) (Table 1). Finally, nondiabetic subjects had different values in all variables except tobacco exposure, platelets, lymphocytes and MPV (Table S1).

### 3.1. Circulating Soluble CD36 in the Study Groups

**Figure 1:**
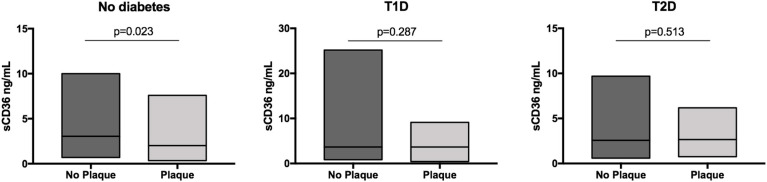
Concentrations of sCD36 according to the presence of atherosclerotic plaque by group. Soluble CD36 in quartiles was Ln transformed.

The median plasma concentration of sCD36 was different between patients with and without atherosclerotic plaques in the whole population (2.58 ng/mL vs. 3.17 ng/mL; *p* = 0.009). In head-to-head comparisons, the concentration of sCD36 was different between nondiabetic subjects with and without atherosclerotic plaques (2.02 ng/mL vs. 3.04 ng/mL; *p* = 0.023). Conversely, there was no difference between T1D patients with and without plaques (3.65 ng/mL vs. 3.66 ng/mL; *p* = 0.287) or between T2D patients with and without atherosclerotic plaques (2.66 ng/mL vs. 2.57 ng/mL; *p* = 0.513) (Figure 1). Furthermore, according to the atherosclerotic plaque burden, sCD36 concentration was different between T2D patients with one plaque vs. multiple plaques (3.44 ng/mL vs. 1.70 ng/mL; *p* = 0.043). Although in the T2D and nondiabetic groups, sCD36 concentrations were higher in echolucent compared to echogenic/mixed plaques, no significant differences were found. In T1D, echogenic/mixed plaques showed non-significantly higher levels of sCD36 (Table S2).

### 3.2. Soluble CD36 as a Marker of Subclinical Carotid Atherosclerosisf

In the logistic regression model of the whole group of study participants, the variables independently associated with the presence of atherosclerotic plaque were older age (OR = 1.084, *p* < 0.001), higher systolic blood pressure (OR = 1.023, *p* < 0.001), tobacco exposure (OR = 2.979, *p* < 0.001), higher BMI (OR = 1.043, *p* = 0.015), T1D (OR = 2.778, *p* < 0.001) and T2D (OR = 2.034, *p* = 0.011) (Table S3). In the nondiabetic control group, the variables independently associated with the presence of atherosclerotic plaque were older age (OR = 1.089, *p* < 0.001), male sex (OR = 2.020, *p* = 0.005), tobacco exposure (OR = 2.404, *p* = 0.001), and higher LDL cholesterol (OR = 1.01, *p* = 0.019) (Table S4). Circulating fatty acid transporter sCD36 was not associated with carotid atherosclerosis when analysed in the whole group or separately in the nondiabetic control group.

**Figure 2:**
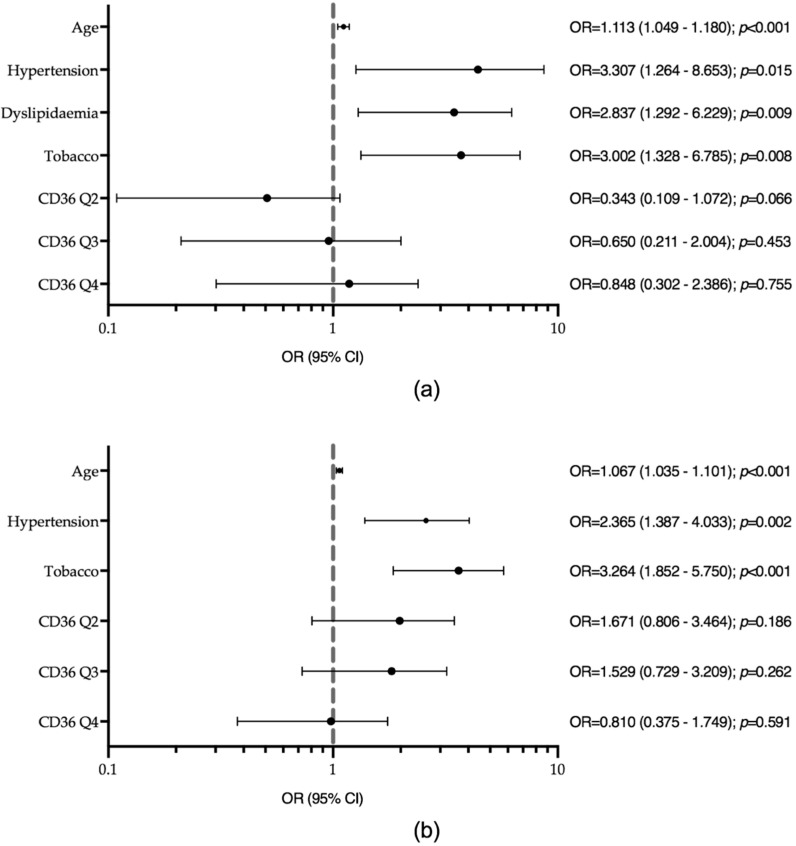
Logistic regression models for the presence of carotid atherosclerotic plaque (**a**) in type 1 diabetes (T1D) (Hosmer and Lemeshow test *p*-value = 0.144). Model adjusted by sex, haematocrit, BMI, haemoglobin and platelets. (**b**) In type 2 diabetes (T2D) (Hosmer and Lemeshow test *p*-value = 0.535). Model adjusted by sex, dyslipidaemia, BMI, haemoglobin, haematocrit and platelets. Tobacco, tobacco exposure; HDL, high-density lipoprotein.

In the logistic regression model for T1D, the variables independently associated with the presence of atherosclerotic plaque were older age (OR = 1.113, *p* < 0.001), hypertension (OR = 3.307, *p* = 0.015), dyslipidaemia (OR = 2.837, *p* = 0.009), and tobacco exposure (OR = 3.002, *p* = 0.008) (Figure 2a). sCD36 was not associated with plaque presence in this group. The inclusion of sCD36 in the model did not provide any discriminative power, i.e., the area under the ROC curve was 0.868, *p* < 0.001 without sCD36 vs. 0.870, *p* < 0.001 with sCD36 (Figure S1a).

In the logistic regression model for T2D, the variables independently associated with the presence of plaque were older age (OR = 1.067, *p* < 0.001), hypertension (OR = 2.365, *p* = 0.002), and tobacco exposure (OR = 3.264, *p* < 0.001), but not sCD36 (Figure 2b). The ROC curve showed no additional discriminative power with the addition of sCD36 to the model: AUCROC 0.740, *p* < 0.001 without sCD36 vs. 0.751, *p* < 0.001 with sCD36 (Figure S1b).

Furthermore, we built a multinomial logistic regression model for carotid atherosclerotic plaque burden, i.e., stratified as no plaque, one plaque, and multiple plaques, and model for intima-media thickness for the whole study group. To avoid model meddling because of the group characteristics, we also analysed the T1D, T2D and nondiabetic control groups separately. The burden of atherosclerotic plaque was not independently associated with the sCD36 quartiles in any of the three groups or the whole study group. The models with the whole group, T1D, T2D and the nondiabetic group can be found in Supplementary material (Tables S5–S8). The intima-media thickness was not independently associated with sCD36 concentrations in any of the three groups or in the whole study group. The results of these models can be found in Supplementary material (Table S9).

### 3.3. Flow-Cytometric Analysis of CD36 and Real-Time PCR

Flow-cytometric analysis was used to detect the expression of CD36 in circulating mononuclear cells from patients with and without atherosclerotic plaques. Patients’ clinical and anthropometric characteristics are shown in Table S10. In the monocytes and leukocyte populations, we did not find significant differences in the expression of the fatty acid transporter scavenger receptor between patients with and without atherosclerotic plaques (Figure S2). No differences in CD36 mRNA expression were found between the patients with and without carotid atherosclerotic plaques (Figure S3).

## 4. Discussion

Taken together, our results show no differences in the fatty acid transporter sCD36 concentration between subjects with and without carotid atherosclerotic plaque in the T1D, T2D or nondiabetic control groups. All of these study groups were free of macrovascular disease and did not have established chronic kidney disease. There was no association of fatty acid transporter with the presence or burden of established subclinical carotid atherosclerosis in any of the groups. We assessed the majority of variables that were previously described to be related to sCD36. Among them are age, sex, blood pressure (systolic and diastolic), tobacco exposure, BMI, lipid profile (HDL, LDL, triglycerides, total cholesterol), haematocrit, haemoglobin, platelets, serum creatinine, blood glucose, HbA1c and medication use. However, the associations observed were between atherosclerotic plaque and classical cardiovascular risk factors, i.e., hypertension, dyslipidaemia, tobacco exposure and age, but not with sCD36.

The fatty acid transporter CD36 is believed to play a role in the development of atherosclerosis through its ability to bind and internalize modified lipids, such as ox-LDL. This action facilitates the formation of macrophage foam cells and their localisation to the subendothelial space, promoting endothelial dysfunction, CV disease and associated metabolic disorders [28,29]. Nevertheless, this process did not appear to be reliably associated with its circulating concentration. Indeed, previous studies in patients with early-onset coronary artery disease have reported that there was no association between fatty acid transporter CD36 genotypes and sCD36 concentrations [14]. Few studies have assessed sCD36 concentrations in patients with atherosclerotic disease, and none have assessed sCD36 concentrations in subjects with T1D. Moreover, to the best of our knowledge, this is the first study to analyse atherosclerosis as represented by the presence and burden of atherosclerotic plaque in patients with T1D, T2D and no diabetes.

Concerning the potential role of sCD36 as a biomarker of cardiovascular disease, we found no association between increased concentration of sCD36 and the presence or burden of carotid atherosclerosis. On the contrary, we found higher sCD36 concentration in nondiabetic subjects without plaque compared with those with plaque. This could suggest a possible protective role of higher sCD36 concentration against subclinical carotid atherosclerosis in nondiabetic subjects. A recent study reported a possible protective effect of sCD36 concentration against metabolic syndrome components in patients with early coronary artery disease [30]. Several studies have analysed sCD36 concentrations in subjects with different atherosclerotic measures. Some of them have reported sCD36 concentrations to be associated with cIMT both in nondiabetic and in diabetic subjects [11,17]. However, it should be mentioned that the accuracy of cIMT as a marker of atherosclerosis is lower than that of the presence of atherosclerotic plaque(s) since cIMT may be influenced by factors that do not necessarily reflect the atherosclerotic process, such as age and hypertension [31,32] Serum sCD36 concentrations have also been analysed in subjects with atherosclerotic plaques by other researchers. Among these studies, Handberg et al. compared sCD36 concentrations in three groups of subjects from the general population, all of them with high-grade internal carotid stenotic plaques [18]. They reported that those subjects with symptomatic plaques (i.e., symptoms within the previous two months) showed higher sCD36 concentrations compared with asymptomatic subjects. They also reported that the increase in sCD36 was restricted to those with most recent symptoms. The atherosclerotic plaques analysed in that study differ from those of subjects in our study in that the latter were asymptomatic and that none of the plaques were stenotic in patients with early onset (<50 years) clinically stable coronary artery disease. In line with our findings, Rac et al. found no relationship between sCD36 concentrations and radiological parameters of atherosclerosis (cIMT and plaque) [33]. Finally, discordant results have been reported on the association between sCD36 concentrations and the risk of incident CV events. In a population-based cohort of Danish subjects, sCD36 was not predictive of coronary risk [34]. On the other hand, another study that investigated subjects with stage 5 chronic kidney disease reported that increased sCD36 concentrations predicted an increased risk of CV mortality [19]. The discordant results of the different studies performed so far on the association between sCD36 concentrations and atherosclerosis could be explained by several reasons. One of them is the heterogeneity of patients included in the different studies, ranging from healthy subjects to those with T2D, chronic kidney disease or even recent CV events. Further, the definition of atherosclerosis varies among studies, including different measures like cIMT, subclinical atherosclerotic plaques or plaques associated with a recent CV event. Finally, these differences might also be explained by the different methods used to quantify sCD36 concentrations.

Therefore, there is a lack of clear answers as to the relationship between plasma sCD36 concentration and carotid atherosclerosis in subjects with T1D or T2D or nondiabetic controls. Many studies agree that there is a lack of reliable, well-characterized or standardized methods to evaluate its concentration [14,35]. Moreover, the pre-analytical protocols do not explain the variability of the different results reported in the published studies [35,36,37]. Thus, discordant results may be due to different causes, including the origin of fatty acid transporter particles in the plasma of patients [38,39] and the management of diabetic patients, which may influence the level of glycated CD36 [38,40]. Previous studies have shown that microparticles (MP), small membranous microvesicles that can be released from any eukaryotic cell, are an important source of plasma CD36. The studies reported that CD36 MP of T2D patients are mainly derived from erythrocytes and those of healthy controls from endothelial cells; moreover, in both group of subjects, platelets are another source of sCD36 [39,41]. Indeed, it has been reported that plasma CD36 MP is a better biomarker for T2D than protein concentration [39]. Therefore, measuring plasma CD36 MP content may improve detection of actual CD36 concentrations. However, in contrast to ELISA, which is a well-established method, to our knowledge no protocol has been developed yet to measure CD36 MP that meets the quality standards required to be used extensively in the clinical practice [42]. Regarding the possible causes mentioned above, we did not find differences in CD36 surface expression on monocytes and leukocytes of peripheral blood among patients with vs. without carotid atherosclerotic plaque. On the other hand, most diabetic patients included in the current study were well controlled, and this could also account for differences among studies. Another condition that could affect CD36 levels is the number of MP with which this molecule is associated, e.g., fatty acid transporter CD36 related to platelet-derived MP may be increased in proportion to these cell particles in subjects without diabetes [39,41]. We previously reported that an increased circulating platelet number is independently associated with a moderate-to-high sCD36 concentration in non-diabetic subjects [36].

Our study has some limitations that merit consideration. First, the cross-sectional study design excludes any conclusions about causality. Thus, a large prospective study is necessary to establish the usefulness of the sCD36 plasma concentration as a predictive factor for cardiovascular disease. Second, we adjusted for risk factors known to be related to atherosclerosis; however, the possibility that some other confounding factors can influence may have been incompletely accounted for. Third, in the flow-cytometric analysis of CD36 and in CD36 mRNA expression few samples were included. Finally, CD36 was not assessed in platelets, which are an important source of this molecule.

## 5. Conclusions

Circulating plasma sCD36 concentration does not appear to be a biomarker of the presence or burden of subclinical nonstenotic carotid atherosclerosis. The possibility that other markers related to the CD36 protein such as CD36-associated MP as well as its main cellular source could be a biomarker of subclinical carotid atherosclerosis should be further explored. In addition, the measurement of sCD36 concentrations must be improved through the use of methodologies that allow for evaluating all circulating sources of this molecule. The use of assays with a higher sensitivity to detect sCD36 could contribute to assess more accurately the role that sCD36 actually plays as a marker of subclinical atherosclerosis. Thus, our study does not eliminate the potential of sCD36 as a biomarker of future atherosclerotic cardiovascular events. Prospective studies that include subjects with and without carotid atherosclerosis may help in clarifying this question.

## Figures and Tables

**Table 1:** Clinical and anthropometrical characteristics of the type 1 and type 2 diabetes groups by the presence or absence of atherosclerotic plaques.

	Type 1 Diabetes			Type 2 Diabetes		
	No Plaque	Plaque	*p*	No Plaque	Plaque	*p*
*n*	149	76		112	164	
Sex, men	69 (46.3%)	40 (52.6%)	0.449	53 (47.3%)	90 (54.9%)	0.267
Age, years	41.0 [36.0;47.0]	51.0 [45.8;59.2]	<0.001	56.5 [48.0;64.0]	61.0 [54.0;68.0]	<0.001
BMI, Kg/m^2^	24.7 [22.1;27.9]	26.1 [23.9;28.1]	0.014	30.4 [28.3;34.7]	30.0 [27.3;34.3]	0.143
Obesity	21 (14.3%)	15 (19.7%)	0.392	63 (56.8%)	83 (50.6%)	0.379
Alcohol, g/day	2.25 [0.00;7.25]	2.65 [0.02;6.98]	0.749	0.56 [0.00;8.27]	3.03 [0.00;11.2]	0.029
Tobacco	67 (45.0%)	49 (64.5%)	0.009	48 (42.9%)	101 (61.6%)	0.003
sBP, mmHg	124 (15.6)	135 (17.0)	<0.001	136 (17.7)	142 (19.4)	0.006
dBP, mmHg	74.3 (9.32)	75.0 (10.6)	0.611	77.8 (10.3)	75.9 (10.7)	0.153
Hypertension	17 (11.4%)	38 (50.0%)	<0.001	48 (42.9%)	105 (64.0%)	0.001
Dyslipidaemia	41 (27.5%)	55 (72.4%)	<0.001	48 (42.9%)	81 (49.4%)	0.344
Statins	64 (43.0%)	32 (42.1%)	1.000	40 (35.7%)	76 (46.3%)	0.103
Antiplatelet	28 (18.8%)	35 (46.1%)	<0.001	35 (31.2%)	64 (39.0%)	0.232
Glucose, mg/dL	162 [118;206]	146 [98.0;209]	0.308	147 [116;198]	148 [119;175]	0.576
Creatinine, mg/dL	0.76 (0.15)	0.79 (0.16)	0.262	0.77 [0.68;0.90]	0.80 [0.69;0.94]	0.353
ALT, mg/dL	16.0 [12.0;20.0]	18.0 [14.0;25.5]	0.005	19.0 [16.0;30.0]	20.0 [16.0;27.0]	0.703
Triglycerides, mg/dL	64.0 [49.0;85.0]	69.5 [55.0;92.0]	0.086	120 [84.5;175]	114 [84.8;157]	0.254
T Cholesterol, mg/dL	184 (28.2)	178 (28.6)	0.191	186 (38.2)	186 (35.6)	0.979
HDL, mg/dL	64.0 [54.2;75.0]	58.5 [52.8;71.2]	0.120	46.0 [39.0;57.0]	49.0 [42.5;60.0]	0.051
LDL, mg/dL	103 [87.0;119]	98.0 [83.0;111]	0.205	109 [88.5;131]	108 [88.4;126]	0.613
HbA1c, %	7.30 [6.90;7.80]	7.60 [7.00;8.20]	0.122	7.70 [6.85;8.70]	7.50 [6.80;8.30]	0.321
Haemoglobin, g/dL	14.0 (1.21)	14.0 (1.45)	0.900	13.8 (1.45)	13.7 (1.30)	0.695
Haematocrit, %	41.5 (3.31)	41.5 (3.89)	0.910	41.2 (3.65)	41.1 (3.47)	0.857
Platelets, ×10^9^/L	225 [200;255]	221 [189;259]	0.513	220 [190;267]	227 [186;278]	0.611
Lymphocytes, ×10^9^/L	1.90 [1.50;2.31]	1.83 [1.44;2.21]	0.375	1.99 [1.67;2.46]	2.06 [1.62;2.52]	0.562
MPV, fL	9.70 [9.00;10.5]	9.75 [9.17;10.3]	0.841	9.20 [8.75;9.70]	9.40 [9.05;9.90]	0.020
IMT	0.63 [0.57;0.70]	0.63 [0.57;0.69]	0.670	0.73 [0.67;0.81]	0.84 [0.73;0.92]	<0.001

Mean (SD) or median [Interquartile range are used for continuous variables]; frequency (percentage) is used for categorical variables. Soluble CD36 in quartiles was Ln transformed.

## Supplementary Materials

The following are available online at https://www.mdpi.com/2077-0383/9/6/1700/s1, Table S1: Soluble CD36 concentrations in the study groups according to plaque type characteristics, Table S2: Logistic regression model for the presence of atherosclerotic plaque in the whole group. Table S3: Logistic regression model for atherosclerotic plaque in the non-diabetic control group. Table S4: Multinomial regression model for the burden of atherosclerotic plaque in the whole group. Table S5: Multinomial regression model for the burden of atherosclerotic plaque in the type 1 diabetes group. Table S6: Multinomial regression model for the burden of atherosclerotic plaque in the type 2 diabetes group. Table S7: Multinomial regression model for the burden of atherosclerotic plaque in the control group. Table S9: Multinomial regression models for the intima-media thickness in the study groups. Table S10: Clinical and anthropometrical characteristics of flow cytometric and Real-time PCR analysis. Figure S1: Receiver operating characteristic (ROC) curve showing the relationship between sensitivity and 1-specificity in determining the discriminatory ability of the logistic regression model with and without sCD36 as a predictor for (a) plaque presence in T1D and (b) plaque presence in T2D. Figure S2: Ex vivo flow-cytometric analysis of CD36 from subjects with and without atherosclerotic plaque. (a) CD36 median fluorescence intensity (MFI) in the monocyte population. (b) CD36 median fluorescence intensity (MFI) in the leukocyte population. Mann-Whitney test *p* = 0.356 and *p* = 458, respectively. Figure S3: CD36 mRNA expression was not different between subjects with and without atherosclerotic plaque. CD36 mRNA expression was analysed by real-time PCR and normalized to GAPDH. The data show the mean fold change relative to subjects with and without atherosclerotic plaque. Mann-Whitney test *p* − 0.551.
