# Supplementary material for: The Circulating Fatty Acid Transporter Soluble CD36 Is Not Associated with Carotid Atherosclerosis in Subjects with Type 1 and Type 2 Diabetes Mellitus
Source: J Clin Med. 2020 Jun 2;9(6):1700. doi: 10.3390/jcm9061700 (PMC7355534; doi:10.3390/jcm9061700)
Supplement: Supplementary file 1 [file jcm-09-01700-s001.pdf]

Table S1. Clinical and anthropometrical characteristics of the Type 1 and type 2 diabetes groups by the presence or absence of atherosclerotic plaque.

	**No plaque**	**Plaque**	**p overall**
n	386	136	
Sex, men	137 (35.5%)	75 (55.1%)	**<0.001**
Age, years	45.0 [37.0;54.0]	58.0 [50.0;67.0]	**<0.001**
BMI, Kg/m^2	25.0 [22.6;27.3]	27.2 [25.2;30.0]	**<0.001**
Obesity	49 (13.0%)	33 (25.0%)	**0.002**
Alcohol, g/day	2.62 [0.00;9.79]	4.99 [0.00;22.4]	**0.010**
Tobacco	192 (49.7%)	78 (57.4%)	0.153
sBP, mmHg	118 [108;127]	130 [122;138]	**<0.001**
dBP, mmHg	75.0 [69.0;82.0]	81.0 [74.0;86.2]	**<0.001**
Hypertension	46 (12.2%)	35 (26.7%)	**<0.001**
Dyslipidaemia	73 (19.5%)	42 (31.6%)	**0.006**
Statins	72 (19.2%)	42 (31.6%)	**0.005**
Glucose, mg/dL	88.0 [83.0;95.0]	92.5 [86.0;102]	**<0.001**
Creatinine, mg/dL	0.78 [0.69;0.92]	0.83 [0.72;0.96]	**0.028**
ALT, mg/dL	16.0 [12.0;23.0]	18.0 [14.8;22.2]	**0.028**
Triglycerides, mg/dL	85.0 [64.0;121]	95.0 [74.8;136]	**0.001**
T Cholesterol, mg/dL	194 [172;223]	204 [183;233]	**0.001**
HDL, mg/dL	58.0 [50.0;69.0]	54.0 [46.0;67.0]	**0.026**
LDL, mg/dL	116 [97.0;139]	128 [108;152]	**0.001**
HbA1c, %	5.40 [5.20;5.70]	5.60 [5.30;5.80]	**0.001**
Haemoglobin, g/dL	14.0 [13.2;15.0]	14.6 [13.8;15.5]	**<0.001**
Haematocrit, %	42.0 (3.60)	43.6 (3.28)	**<0.001**
Platelets, x10*9/L	236 [206;274]	232 [200;272]	0.075
Lymphocytes, x10*9/L	2.11 [1.72;2.52]	2.13 [1.81;2.67]	0.083
MPV, fL	9.80 [9.25;10.7]	9.90 [9.20;10.8]	0.674
IMT	0.65 [0.59;0.73]	0.77 [0.67;0.85]	**<0.001**

Mean (SD) or median [Interquartile range] for continuous variables and frequency (percentage) for categorical variables. Soluble CD36 in quartiles was Ln transformed.

Table S2. Soluble CD36 concentrations in the study groups according to plaque type characteristic.

	No plaque	Echolucent	Echogenic/Mixed	p.overall	p.Echolucent vs Echogenic/Mixed
**All groups**					
n	623	164	200		
sCD36 ng/mL	3.15 [0.68;10.4]	2.95 [0.67;8.43]	2.17 [0.40;5.88]	**0.015**	0.066
**Control**					
n	374	73	58		
sCD36 ng/mL	2.93 [0.69;9.51]	3.70 [0.48;8.33]	1.49 [0.06;5.44]	**0.016**	0.050
**Type 1 diabetes**					
n	137	33	37		
sCD36 ng/mL	4.34 [0.76;18.4]	2.77 [0.72;13.2]	2.90 [0.51;8.76]	0.714	0.750
**Type 2 diabetes**					
n	112	58	105		
sCD36 ng/mL	2.57 [0.60;9.70]	2.71 [0.90;7.89]	2.65 [0.65;5.56]	0.419	0.432

Median [Interquartile range].

Table S3. Logistic regression model for the presence of atherosclerotic plaque in the whole group.

	OR	95% CI	p
Dyslipidaemia	1.335	0.954 – 1.869	0.092
Type 1 diabetes	2.778	1.573 – 4.906	<0.001
Type 2 diabetes	2.034	1.178 – 3.513	0.011
Body mass index	1.043	1.008 – 1.080	0.015
Tobacco exposure	2.979	2.115 – 4.196	<0.001
Systolic Blood Pressure	1.023	1.013 – 1.034	<0.001
HbA1c	0.847	0.712 – 1.008	0.061
Age	1.084	1.066 – 1.102	<0.001
sCD36 Quartile 2	0.989	0.627 – 1.559	0.962
sCD36 Quartile 3	1.328	0.850 – 2.074	0.213
sCD36 Quartile 4	0.847	0.533 – 1.347	0.484

In logistic regression model sCD36 was Ln transformed. Model adjusted by alcohol, sex, statins, diastolic blood pressure, creatinine, eGFR, ALT, triglycerides and HDL cholesterol. Hosmer and Lemeshow test p-value=0.167. BMI, body mass index.

Table S4. Logistic regression model for atherosclerotic plaque in the non-diabetic control group.

	OR	95% CI	p
Age	1.089	1.066 – 1.114	<0.001
Sex, woman	0.495	0.304 – 0.804	0.005
Body mass index	1.056	0.998 – 1.116	0.059
Tobacco exposure	2.404	1.432 – 4.036	0.001
LDL cholesterol	1.01	1.002 – 1.018	0.019
Glucose	1.02	0.997 – 1.044	0.085
sCD36 Quartile 2	1.023	0.511 – 2.050	0.949
sCD36 Quartile 3	1.688	0.866 – 3.291	0.124
sCD36 Quartile 4	1.088	0.53 – 2.232	0.819

In logistic regression model sCD36 was Ln transformed. Model adjusted by alcohol, hypertension, dyslipidaemia, creatinine, eGFR, triglycerides, HDL cholesterol, LDL cholesterol, haemoglobin, haematocrit, platelets and lymphocytes. Hosmer and Lemeshow test p-value=0.695. BMI, body mass index.

Table S5. Multinomial regression model for the burden of atherosclerotic plaque in the whole group.

	One plaque			Multiple plaques		
	**OR**	**95% CI**	**p**	**OR**	**95% CI**	**P**
Age	1.072	1.052 – 1.091	**<0.001**	1.114	1.089 – 1.138	**<0.001**
Sex, woman	0.761	0.522 – 1.109	0.155	0.502	0.326 – 0.773	**0.002**
Hypertension	1.584	1.043 – 2.40	**0.031**	2.252	1.455 – 3.487	**<0.001**
Dyslipidaemia	1.148	0.784 – 1.680	0.478	1.856	1.235 – 2.791	**0.003**
Body mass index	1.049	1.010 – 1.089	**0.013**	1.020	0.977 – 1.065	0.372
Diabetes type 1	2.262	1.394 – 3.671	**<0.001**	2.127	1.192 – 3.797	**0.011**
Diabetes type 2	1.497	0.937 – 2.390	0.091	1.838	1.124 – 3.005	**0.015**
Tobacco	2.232	1.502 – 3.318	**<0.001**	3.392	2.135 – 5.387	**<0.001**
sCD36 Quartile 2	1.138	0.690 – 1.875	0.612	0.953	0.541 – 1.638	0.861
sCD36 Quartile 3	1.454	0.884 – 2.392	0.140	1.283	0.751 – 2.191	0.362
sCD36 Quartile 4	0.939	0.558 – 1.581	0.814	0.803	0.453 – 1.424	0.453

In multinomial regression model sCD36 was Ln transformed.

Table S6. Multinomial regression model for the burden of atherosclerotic plaque in the type 1 diabetes group.

	One plaque			Multiple plaques		
	**OR**	**95% CI**	**p**	**OR**	**95% CI**	**P**
Age	1.100	1.045 – 1.158	**<0.001**	1.115	1.082 – 1.223	**<0.001**
Hypertension	2.165	0.823 – 5.691	0.117	6.378	2.159 – 18.84	**<0.001**
Dyslipidaemia	3.514	1.526 – 8.091	**0.003**	2.286	0.804 – 6.503	0.121
Tobacco	2.326	1.004 – 5.391	**0.049**	5.022	1.664 – 15.16	**0.004**
sCD36 Quartile 2	0.422	0.137 – 1.298	0.132	0.647	0.147 – 2.855	0.566
sCD36 Quartile 3	0.662	0.210 – 2.084	0.481	1.131	0.317 – 5.377	0.711
sCD36 Quartile 4	0.662	0.229 – 1.917	0.447	1.764	0.448 – 6.954	0.417

In multinomial regression model sCD36 was Ln transformed.

Table S7. Multinomial regression model for the burden of atherosclerotic plaque in the type 2 diabetes group.

	One plaque			Multiple plaques		
	**OR**	**95% CI**	**p**	**OR**	**95% CI**	**P**
Age	1.034	0.998 – 1.070	0.060	1.112	1.068 – 1.157	**<0.001**
Sex, women	1.482	0.712 – 3.083	0.292	1.041	0.472 – 2.296	0.921
Hypertension	1.825	0.975 – 3.415	0.060	3.319	1.695 – 6.499	**<0.001**
Tobacco	2.446	1.151 – 5.195	**0.020**	6.273	2.706 – 14.54	**<0.001**
DM Treat OAD+Ins	0.644	0.293 – 1.417	0.274	1.194	0.580 – 2.555	0.647
DM Treat Insulin	1.405	0.404 – 4.892	0.593	1.301	0.338 – 5.014	0.702
DM Treat Others	0.894	0.369 – 2.172	0.806	0.174	0.043 – 0.705	**0.014**
sCD36 Quartile 2	2.312	0.921 – 5.805	0.074	1.179	0.497 – 2.799	0.708
sCD36 Quartile 3	2.314	0.913– 5.863	0.077	0.843	0.348– 2.039	0.704
sCD36 Quartile 4	1.546	0.599 – 3.996	0.366	0.410	0.151 – 1.116	0.081

In multinomial regression model sCD36 was Ln transformed.

Table S8. Multinomial regression model for the burden of atherosclerotic plaque in the control group.

	One plaque			Multiple plaques		
	**OR**	**95% CI**	**p**	**OR**	**95% CI**	**P**
Age	1.067	1.042 – 1.093	**<0.001**	1.095	1.066 – 1.126	**<0.001**
Sex, woman	0.491	0.282– 0.857	**0.012**	0.262	0.139 – 0.492	**<0.001**
Hypertension	1.133	0.558 – 2.304	0.729	0.922	0.424 – 2.004	0.838
Dyslipidaemia	0.880	0.463 – 1.673	0.697	1.696	0.893 – 3.218	0.106
Body mass index	1.073	1.007 – 1.144	**0.030**	1.076	0.998 – 1.159	0.054
sCD36 Quartile 2	1.160	0.542– 2.481	0.702	0.716	0.294 – 1.741	0.461
sCD36 Quartile 3	1.664	0.786 – 3.526	0.183	1.437	0.628 – 3.289	0.391
sCD36 Quartile 4	0.879	0.386 – 2.001	0.759	1.099	0.473– 2.554	0.825

In multinomial regression model sCD36 was Ln transformed.

Table S9. Multinomial regression models for the intima-media thickness in the study groups.

	sCD36 Quartile 2	sCD36 Quartile 3	sCD36 Quartile 4	Adjusted variables
**T2D**	4.79 (0.19-1.19)<br>0.339	9.32(0.37-237.1)<br>0.176	3.02(0.09-97.9)<br>0.533	Age, sex, plateletsQ4, antiplatelets, DLP, MPV, DM-Treat
**T1D**	1.18(2.12-6.62)<br>**0.020**	4.31(7.20-2.58)<br>0.071	0.21(0.00-10.3)<br>0.434	Age, sex, BMI, tobaccoExp, Total cholesterol, LDL
**No diabetes**	4.71(0.79-27.9)<br>0.087	4.23(0.74-24.2)<br>0.105	0.70(0.115-4.22)<br>0.697	Age, sex, HT, BMI, Triglycerides, HbA1c, PlateletsQ4
**Whole group**	4.72(0.29-76.1)<br>0.273	2.58(0.16-41.7)<br>5.028	0.55(0.35-8.54)<br>0.671	Age, sex, HT, TobaccoExp, BMI, ALT, diabetes, Triglycerides, PlateletsQ4, LDL, MPV

In multinomial regression model sCD36 was Ln transformed. ALT, alanine transaminase; BMI, body mass index; DLP, dyslipidaemia; DM-Treat, diabetes treatment; HT, hypertension; IMT, intima media thickness; LDL, low density lipoprotein; MPV, mean platelet volume.

Table S10. Clinical and anthropometrical characteristics of flow cytometric and Real-time PCR analysis.

	**No diabetes**	**T2D**	**p.overall**
n	28	22	
Sex, men	14 (50.0%)	9 (40.9%)	0.723
Age, years	51.2 (7.02)	60.3 (8.07)	<0.001
Hypertension	5 (17.9%)	14 (63.6%)	0.003
Dyslipidaemia	3 (10.7%)	16 (72.7%)	<0.001
BMI, Kg/m2	24.7 [23.3;26.7]	28.9 [27.0;31.6]	<0.001
Waist, cm	89.8 (10.3)	103 (12.4)	<0.001
Smoking:			0.220
No	13 (46.4%)	8 (36.4%)	
Yes	2 (7.14%)	6 (27.3%)	
Former smoker	13 (46.4%)	8 (36.4%)	
sBP, mmHg	116 (14.2)	135 (16.3)	<0.001
dBP, mmHg	73.1 (10.9)	74.2 (10.8)	0.738
Antiplatelet	0 (0.00%)	7 (31.8%)	0.002
Statins	2 (7.14%)	16 (72.7%)	<0.001
Glucose, mg/dL	89.5 [81.0;93.0]	138 [114;155]	<0.001
Creatinine, mg/dL	0.82 (0.15)	0.74 (0.19)	0.114
Triglycerides, mg/dL	90.5 [78.8;128]	132 [117;197]	0.003
Total_cholesterol, mg/dL	209 (30.9)	179 (36.6)	0.004
HDL_cholesterol, mg/dL	58.8 (10.8)	42.8 (10.6)	<0.001
LDL_cholesterol, mg/dL	130 (30.2)	107 (34.1)	0.021
HbA1c, %	5.40 [5.12;5.60]	7.20 [6.53;7.60]	<0.001
Hemoglobin, g/dL	14.2 (1.21)	14.2 (1.43)	0.941
Hematocrit, %	42.0 (3.47)	42.5 (3.97)	0.659
Platelets, x10*9/L	210 [190;222]	214 [186;245]	0.587
IMT	0.58 [0.52;0.71]	0.68 [0.58;0.74]	0.178
Plaque:			0.035
No	20 (71.4%)	8 (36.4%)	
One plaque	4 (14.3%)	5 (22.7%)	
Multiple plaques	4 (14.3%)	9 (40.9%)	

Mean (SD) or median [Interquartile range] for continuous variables and frequency (percentage) for categorical variables. BMI, body mass index; sBP, systolic blood pressure; dBP, diastolic blood pressure; HDL, high density lipoprotein; LDL, low density lipoprotein; IMT, intima media thickness.

Plaque model in T1D

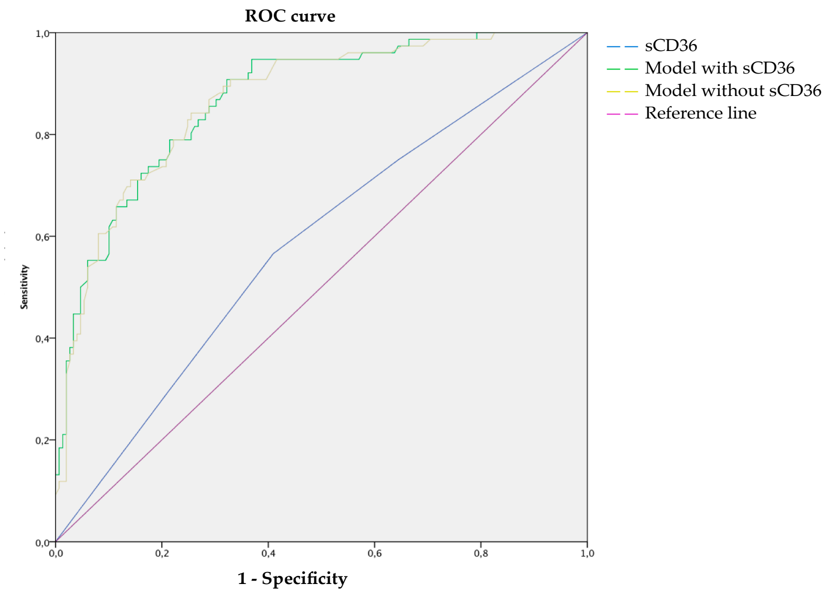

Test Results Variables	Area	Std. Error	Asymptotic Sig.	Asymptotic 95% Confidence Interval	
				Lower Bound	Upper Bound
sCD36	0.582	0.04	0.044	0.503	0.661
Model with sCD36	0.870	0.024	<0.001	0.822	0.917
Model without sCD36	0.868	0.025	<0.001	0.819	0.916

(a)

Plaque model in T2D

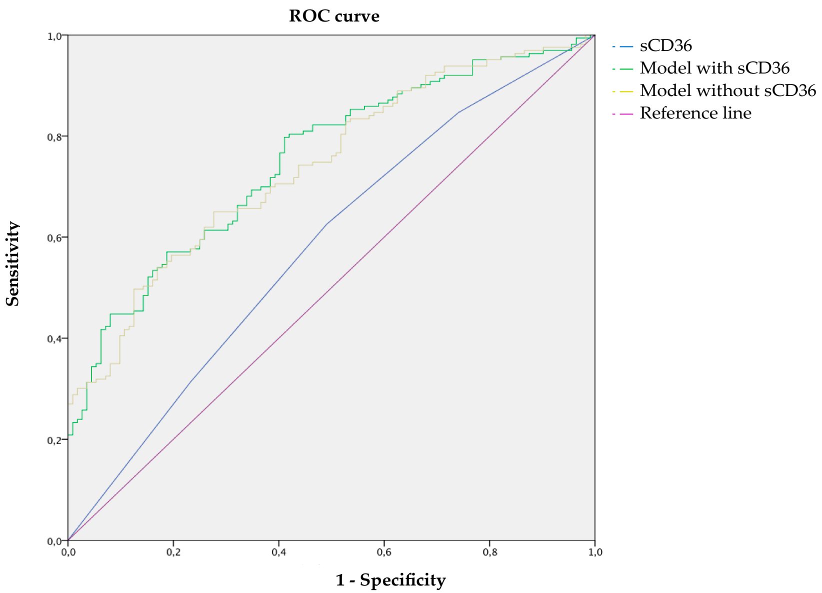

Test Results Variables	Area	Std. Error	Asymptotic Sig.	Asymptotic 95% Confidence Interval	
				Lower Bound	Upper Bound
sCD36	0.581	0.035	0.023	0.512	0.650
Model with sCD36	0.751	0.029	0	0.695	0.808
Model without sCD36	0.740	0.029	0	0.682	0.797

(b)

Figure S1. Receiver operating characteristics (ROC) curve showing the relationship between sensitivity and 1-specificity in determining the discriminatory ability of the logistic regression model with and without sCD36 as predictor for (a) Plaque presence in T1D; and (b) Plaque presence in T2D.

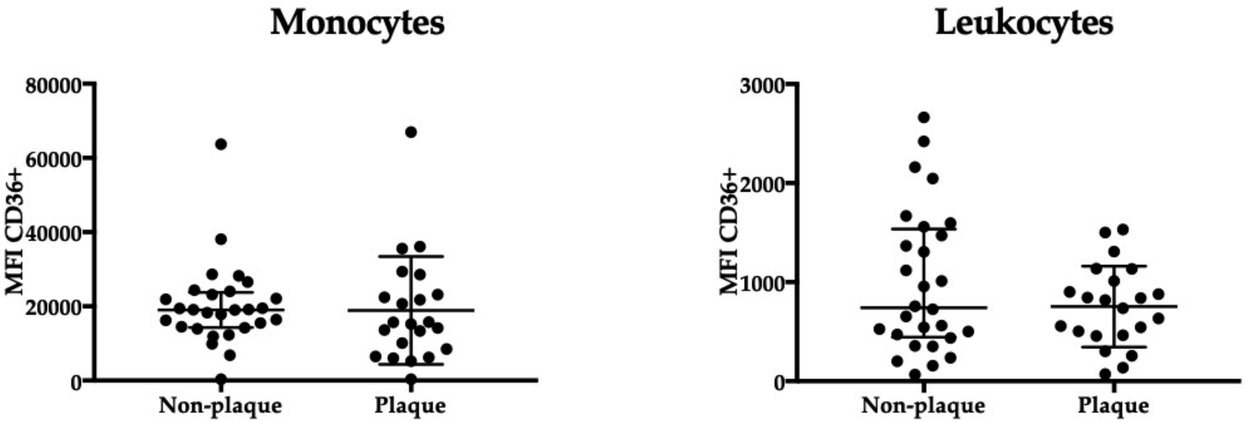

Figure S2. Ex vivo flow cytometric analysis of CD36 from subjects with and without atherosclerotic plaque. (a) CD36 median fluorescence intensity (MFI) in monocyte population. (b) CD36 median fluorescence intensity (MFI) in leukocyte population.

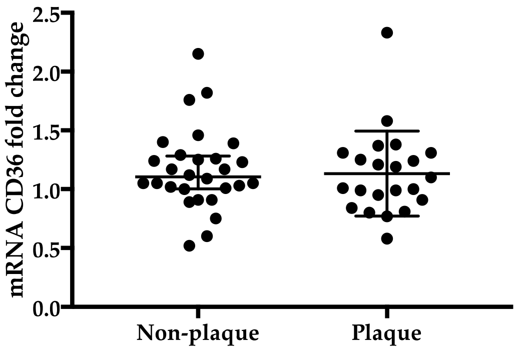

Figure S3. CD36 mRNA expression was not different between T2D and non-diabetic controls. CD36 mRNA expression was analysed by real-time PCR and normalized to GAPDH. The data show the mean of fold change relative control group from the analyses subjects. Mann-Whitney test p=0.232.
